# A lepidic gene signature predicts patient prognosis and sensitivity to immunotherapy in lung adenocarcinoma

**DOI:** 10.1186/s13073-021-01010-w

**Published:** 2022-01-12

**Authors:** Thinh T. Nguyen, Hyun-Sung Lee, Bryan M. Burt, Jia Wu, Jianjun Zhang, Christopher I. Amos, Chao Cheng

**Affiliations:** 1 Institute for Clinical and Translational Research, Baylor College of Medicine, Houston, TX 77030 USA; 2 Division of General Thoracic Surgery, Michael E. DeBakey Department of Surgery, Baylor College of Medicine, Houston, TX 77030 USA; 3 Department of Thoracic/Head and Neck Medical Oncology, The University of Texas MD Anderson Cancer Center, Houston, TX 77030 USA; 4 Department of Imaging Physics, Division of Diagnostic Imaging, The University of Texas MD Anderson Cancer Center, Houston, TX 77030 USA; 5 Department of Genomic Medicine, The University of Texas MD Anderson Cancer Center, Houston, TX 77030 USA; 6 Department of Medicine, Baylor College of Medicine, Houston, TX 77030 USA; 7 Dan L Duncan Comprehensive Cancer Center, Baylor College of Medicine, Houston, TX 77030 USA

**Keywords:** Lung adenocarcinoma, Histology, Tumor immune microenvironment, Gene signature, Prognosis, Genomic alterations, Drug sensitivity, Immunotherapy

## Abstract

**Background:**

Lung adenocarcinoma, the most common type of lung cancer, has a high level of morphologic heterogeneity and is composed of tumor cells of multiple histological subtypes. It has been reported that immune cell infiltration significantly impacts clinical outcomes of patients with lung adenocarcinoma. However, it is unclear whether histologic subtyping can reflect the tumor immune microenvironment, and whether histologic subtyping can be applied for therapeutic stratification of the current standard of care.

**Methods:**

We inferred immune cell infiltration levels using a histological subtype-specific gene expression dataset. From differential gene expression analysis between different histological subtypes, we developed two gene signatures to computationally determine the relative abundance of lepidic and solid components (denoted as the L-score and S-score, respectively) in lung adenocarcinoma samples. These signatures enabled us to investigate the relationship between histological composition and clinical outcomes in lung adenocarcinoma using previously published datasets.

**Results:**

We found dramatic immunological differences among histological subtypes. Differential gene expression analysis showed that the lepidic and solid subtypes could be differentiated based on their gene expression patterns while the other subtypes shared similar gene expression patterns. Our results indicated that higher L-scores were associated with prolonged survival, and higher S-scores were associated with shortened survival. L-scores and S-scores were also correlated with global genomic features such as tumor mutation burdens and driver genomic events. Interestingly, we observed significantly decreased L-scores and increased S-scores in lung adenocarcinoma samples with *EGFR* gene amplification but not in samples with *EGFR* gene mutations. In lung cancer cell lines, we observed significant correlations between L-scores and cell sensitivity to a number of targeted drugs including EGFR inhibitors. Moreover, lung cancer patients with higher L-scores were more likely to benefit from immune checkpoint blockade therapy.

**Conclusions:**

Our findings provided further insights into evaluating histology composition in lung adenocarcinoma. The established signatures reflected that lepidic and solid subtypes in lung adenocarcinoma would be associated with prognosis, genomic features, and responses to targeted therapy and immunotherapy. The signatures therefore suggested potential clinical translation in predicting patient survival and treatment responses. In addition, our framework can be applied to other types of cancer with heterogeneous histological subtypes.

**Supplementary Information:**

The online version contains supplementary material available at 10.1186/s13073-021-01010-w.

## Background

Lung cancer is the leading cause of cancer-related deaths in the USA, causing more than 20% of all cancer deaths in 2020 [1]. Depending on the stage at the time of diagnosis, the prognosis of lung cancer differs substantially among patients. The 5-year survival rate is approximately 55% for localized lung cancer, in contrast to only 5% for distant spread [1]. Of all lung cancer cases, about 85% are non-small cell lung cancer (NSCLC) and 15% are small cell lung cancer [2]. Lung adenocarcinoma, squamous lung carcinoma, and large-cell lung carcinoma are the three major histological types of NSCLC, accounting for 47%, 35%, and 12% of all NSCLC cases, respectively. The application of immune checkpoint blockade therapy (ICBT) has improved the overall prognosis of NSCLC. However, only about 15% of patients have durable benefit from it [3]. The limited response rate to immunotherapy has become a major impediment preventing further prognostic improvement in late-stage NSCLC.

Histology is an important feature of tumors and is associated with major clinical outcomes. Early-stage lung adenocarcinoma can be further divided into five histological subtypes: lepidic, solid, acinar, papillary, and micropapillary [4]. Typically, lung adenocarcinoma is composed of cells with varying histological subtypes, which is known as intratumor heterogeneity. For example, a lung adenocarcinoma tissue may show 50% of papillary, 30% of acinar, and 20% of lepidic patterns at different tumor sub-regions. As such, it has been highly recommended by pathologists to include percentages of histological subtypes during histological review [4]. In general, the lepidic subtype is associated with low-grade tumor; acinar and papillary subtypes are associated with intermediate-grade tumor; solid and micropapillary subtypes are associated with high-grade tumor [5, 6]. The lepidic subtype is associated with good prognosis [5–9], while the solid and micropapillary subtypes are associated with poor prognosis [7, 10–14]. Interestingly, the expression level of PD-L1 varies among different histological subtypes: solid and micropapillary tumors have higher levels than the other subtypes [15–17]. However, it remains unclear how these different histological subtypes vary in their immune microenvironment and whether they show different sensitivity to targeted treatments and immunotherapies.

Several studies have shown that the tumor immune microenvironment (TIME) plays critical roles in the development, progression, and metastasis of cancer [18–20]. In many different cancer types including lung adenocarcinoma, the infiltration levels of immune cells in TIME are associated with patient prognosis [21–25]. In lung adenocarcinoma, T cells seem to be the main type of infiltrating immune cell [26] and the extent of T cell infiltration is positively correlated with patient prognosis [25]. Varn et al. [22] reported that the infiltration of CD8+ T cells and naïve B cells were associated with good prognosis while the infiltration of myeloid cells was associated with poor prognosis in early-stage lung adenocarcinoma. Generally, the presence of immunosuppressive features, including the absence of T cell infiltration and the presence of suppressive macrophages, is associated with poor prognosis [25]. It has also been reported that NSCLC patients with higher PD-L1 protein level tend to have longer survival times when treated by ICBT [27–29]. Nevertheless, the effectiveness of using PD-L1 expression for predicting ICBT response is still under debate [27, 30, 31].

In this study, we analyzed the gene expression data for micro-dissected lung adenocarcinoma tumors with homogenous histological subtypes and found that the five histological subtypes varied dramatically in their immunological features. Additionally, we found the lepidic and solid subtypes had distinguishable gene expression patterns while papillary, micropapillary, and acinar subtypes shared similar expression patterns. According to these observations, we defined two gene expression signatures to characterize lepidic and solid lung adenocarcinoma subtypes, respectively. Given a gene expression profile for a lung adenocarcinoma sample, these two signatures can be used to calculate a lepidic-score (L-score) and a solid-score (S-score) that quantify the relative abundance of lepidic and solid components in the sample. We found that higher L-scores were associated with better prognosis and response to immunotherapy. Moreover, the L-scores of lung cancer cell lines were correlated with their sensitivity to targeted drugs such as EGFR inhibitors. Our analyses revealed critical links between histological composition and clinical outcomes in lung adenocarcinoma.

## Methods

### Dataset collection and processing

The level 3 RNA-seq data of lung adenocarcinoma (LUAD) generated by The Cancer Genome Atlas (TCGA) were retrieved from FireBrowse (http://firebrowse.org) [32]. Processed RNA-seq data of LUAD samples from The National Cancer Institute’s (NCI’s) Clinical Proteomic Tumor Analysis Consortium (CPTAC) were retrieved from NCI’s Genomic Data Commons (GDC) [33, 34]. The large-scale drug sensitivity data generated by the Genomics of Drug Sensitivity in Cancer (GDSC) was retrieved from the GDSC database [35]. Ten microarray datasets used in this study were retrieved from the Gene Expression Omnibus (GEO) [36] with accession numbers GSE58772 (*n* = 48) [37], GSE14814 (*n* = 133) [38], GSE8894 (*n* = 138) [39], GSE13213 (*n* = 117) [40], GSE31210 (*n* = 246) [41], GSE32989 (*n* = 69) [42], GSE93157 (*n* = 65) [43], GSE11969 (*n* = 149) [44], GSE26939 (*n* = 116) [45], and GSE72094 (*n* = 442) [46]. Microarray data from the GEO database provide expression at the probeset level and were transformed into gene-level expression by selecting the probeset with the maximum intensities to represent a gene (for data from one-channel microarray: GSE58772 [37], GSE14814 [38], GSE8894 [39], GSE31210 [41], GSE32989 [42], GSE93157 [43], GSE72094 [46]) or by calculating the average levels of probesets for the same gene (for data from two-channel microarray: GSE13213 [40], GSE11969 [44], GSE26939 [45]). Processed RNA-seq gene expression data represented as Transcript Per Million and clinical data from Bachereau et al. [47] were retrieved from the European Genome-phenome Archive with accession number EGAS00001004343.

TCGA somatic mutation and copy number variation data were also downloaded from FireBrowse (http://firebrowse.org). Only non-silent mutations from somatic mutation data were indicated in the analyses. Some genomic features such as non-synonymous mutation rate and homologous recombination pathway defect score of TCGA LUAD samples were retrieved from a published article by Thorsson et al. [48].

The Zabeck dataset GSE58772 [37] was used to perform immune infiltration analysis and to define gene signatures for lepidic and solid histological subtypes. The data contains expression profiles for 10 acinar, 10 solid, 10 lepidic, 9 micropapillary, and 9 papillary tumor samples purified by microdissection. Zhu dataset GSE14814 [38], TCGA LUAD [32], and CPTAC LUAD [33] were used to validate the signatures with provided histological subtype information. The datasets TCGA LUAD [32], GSE8894 [39], GSE13213 [40], and GSE31210 [41], with considerable number of patients (*n* > 60) with high-quality patient survival times and statuses, were used for prognostic analysis. These data have survival information in the form of either overall survival (TCGA LUAD and GSE13213) or recurrence-free survival (GSE8894 and GSE31210). With a high number of patients and available somatic mutation and copy number variation data, TCGA LUAD [32] was used to investigate the association of signatures with genomic features. Additionally, datasets GSE11969 [44], GSE26939 [45], and GSE72094 [46] were used to validate the findings, with clinical information regarding *TP53*/*EGFR* mutation status for each patient. Regarding the association of signatures with drug sensitivity of cancer cell lines, datasets GSE32989 [42] and GDSC [35] with corresponding information were utilized. Lastly, to investigate the association of signatures with patient response to immunotherapy, Prat dataset GSE93157 [43] and Banchereau dataset [47] were implemented. The former contains 22 adenocarcinoma and 13 squamous cell carcinoma patients treated with pembrolizumab and nivolumab, and the latter contains 81 non-small cell lung cancer patients treated with atezolizumab, providing treatment outcome information for each patient.

In the analyses, only lung adenocarcinoma samples/cell lines included in these datasets were used unless specified. Details about all datasets, including the number of samples/cell lines, descriptions, and relevant publications, are included in Additional file 1: Table S1.

### Computational inference of immune cell infiltration in tumor samples

We applied the previously published BASE computational algorithm [21, 22, 49, 50] to infer the infiltration level of immune cells in tumor samples based on their gene expression profiles. This algorithm estimates the infiltration levels of immune cells by examining the expression levels of immune-cell-specific genes. Full details of the algorithm have been described in a prior publication [22]. In this study, we calculated the infiltration scores of six different immune cell types including naive B cells, memory B cells, CD8+ T cells, CD4+ T cells, NK cells, and myeloid cells.

### Differential gene expression analysis between different histological subtypes

We identified genes that are specifically expressed in each of the five histological subtypes using the Zabeck dataset [37]. For each subtype, we divided samples into two groups with one containing samples of this subtype and the other containing the remaining samples in the dataset. The expression of all genes was compared between the two groups by using the Student’s *t*-test. The resulting *p*-values were adjusted for multiple tests using the Benjamini-Hochberg method. Principal component analysis (PCA) was based on the expression of 1000 genes, which were obtained by merging the top 200 most specific genes (it may not be significant) for each subtype.

### Defining gene signatures to characterize lepidic and solid histological subtypes

Here we use the lepidic subtype as the example. From the above-described differential expression analysis, we have determined the *t*-score and *p*-value for all genes by comparing lepidic samples with all other samples using Student’s *t*-test, which resulted in two vectors {t} and {p}. Based on them, we defined a pair of weighted profiles, denoted as ${w}^{+}=\left({w}_1^{+},{w}_2^{+},\dots, {w}_g^{+}\right)$ and ${w}^{-}=\left({w}_1^{-},{w}_2^{-},\dots, {w}_g^{-}\right)$. For gene *k* with a *t*-score of *t*_*k*_ and *p*-value of *p*_*k*_, ${w}_k^{+}$ and ${w}_k^{-}$ were obtained by (i) calculating −*I*(*t*_*k*_ > 0) log *p*_*k*_ and −*I*(*t*_*k*_ < 0) log *p*_*k*_, (ii) trimming at 10 to avoid extreme values, and (iii) rescaling the values to [0, 1]. The *w*^+^ and *w*^−^ form the gene signature for the lepidic subtype with genes that are more up-regulated and down-regulated being assigned larger values in *w*^+^ and *w*^−^, respectively. The gene signature for solid subtype can be defined similarly.

### Calculation of lepidic-scores and solid-scores in tumor samples

The defined gene signatures were then used to calculate sample-specific L/S-scores to measure the lepidic/solid components in lung adenocarcinoma samples. To this end, we applied a rank-based method similar to the gene set enrichment analysis (GSEA) [51, 52]. First, given the expression profile for a tumor sample, we sorted all genes in decreasing order of expression level to obtain a ranked expression vector *e* = (*e*_*1*_, *e*_*2*_, ..., *e*_*n*_). Second, we examined whether genes with high weight in *w* (*w*^+^ or *w*^−^) had a skewed distribution in {*e*}, and quantified skewness by comparing a foreground function *f(i)* with a background function *b(i).*

$$ f(i)=\frac{\sum_{k=1}^i\left|{e}_k{w}_k\right|}{\sum_{k=1}^n\left|{e}_k{w}_k\right|},1\le i\le n $$

$$ b(i)=\frac{\sum_{k=1}^i\left|{e}_k\left(1-{w}_k\right)\right|}{\sum_{k=1}^n\left|{e}_k\left(1-{w}_k\right)\right|},1\le i\le n $$

While *b(i)* represents a random background distribution, *f(i)* captures the skewed distribution of highly informative genes in a gene expression profile. For example, if genes with high weights tend to have higher expression, *f(i)* will increase more rapidly than *b(i)*. Third, we calculated the maximum deviation between *f(i)* and *b(i)* and normalized it against a null distribution estimated from permuted data, resulting in pairs of scores, *S*^+^ and *S*^−^ from *w*^+^ and *w*^−^, respectively. Fourth, the final score for this sample was calculated as *S* = *S*^+^ − *S*^−^. A higher L- or S-score indicates a higher relative abundance of lepidic or solid components in a tumor.

### Association of L-scores and S-scores with genomic features

We used the following steps to identify genes with mutation status correlated with L-scores and S-scores of samples. First, we selected all genes that were mutated in at least 20 tumor samples in TCGA LUAD [32]. Second, for each of the selected genes, we defined two sample groups based on the mutation status of this gene. Third, we conducted the Wilcoxon rank sum test to compare the L- or S-scores of the two groups. Genes with *p* < 0.01 were selected as significant genes. A similar procedure was used to identify genes with amplification/deletion status correlated with L- and S-scores. The somatic mutation, amplification, and deletion information of genes in TCGA LUAD [32] were downloaded from FireBrowse (http://firebrowse.org).

### Statistical analysis

The R package “*survival*” was implemented to perform survival analyses. Survival distributions between two patient groups were compared using the log-rank test through the “survdiff” function. Univariate and multivariate Cox regression models were performed using the “coxph” function. Wilcoxon rank sum test and Student’s *t*-test were performed for comparing the values between two groups of samples through the R function “wilcox.test” and “t.test,” respectively. Multiple test correction was performed by using the Benjamini-Hochberg method to obtain adjusted *p*-values in the form of false discovery rate (FDR). *p*-values less than 0.05 are considered statistically significant, if stated otherwise. All statistical analyses in this study were conducted using the R software.

## Results

### Different histological subtypes of lung adenocarcinoma vary substantially in the tumor immune microenvironment

**Fig. 1:**
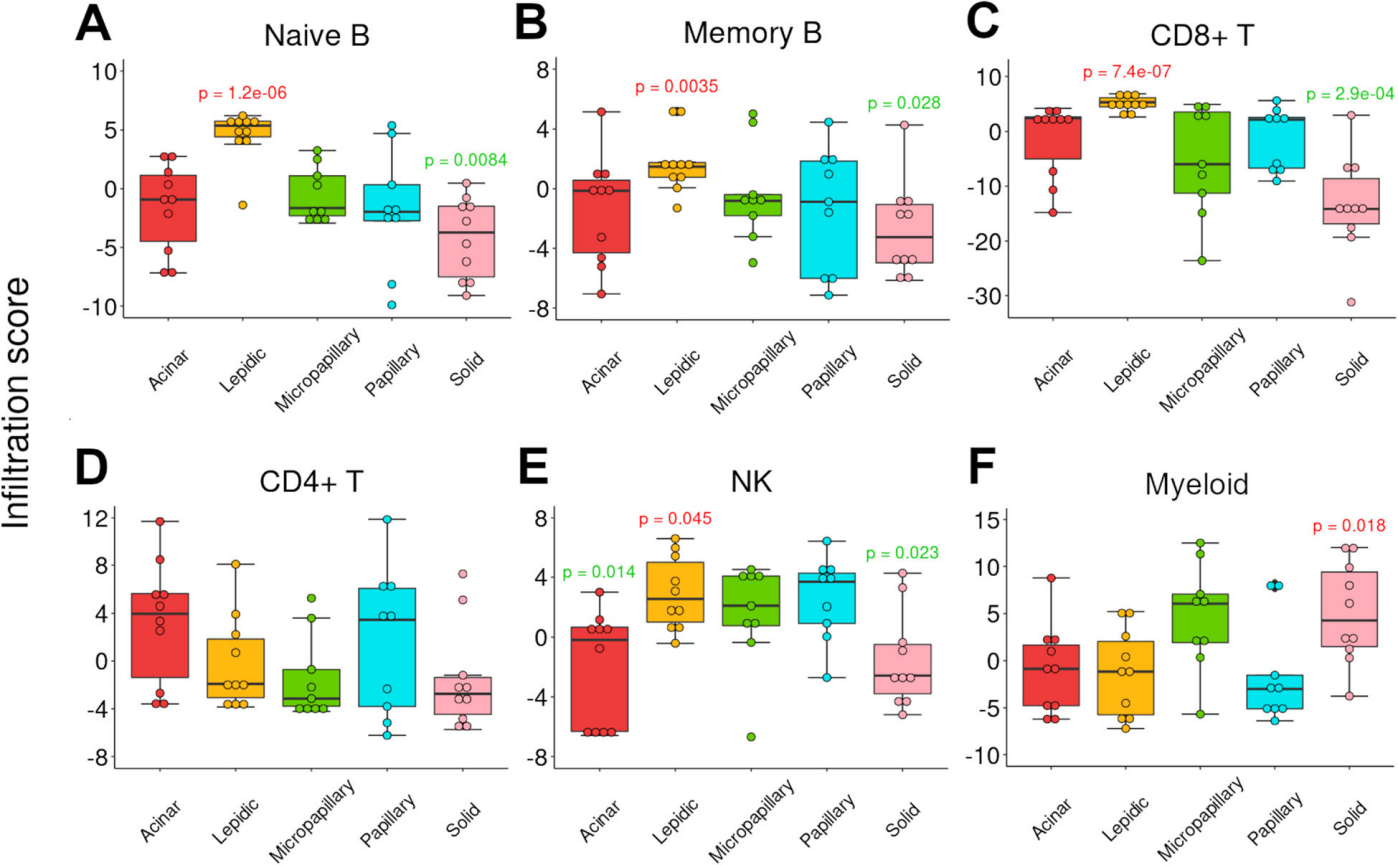
The infiltration levels of immune cells vary significantly between different histological subtypes of lung adenocarcinoma. Boxplots showing the infiltration levels of **A** Naive B, **B** Memory B, **C** CD8+ T, **D** CD4+ T, **E** NK, and **F** myeloid in five different lung adenocarcinoma histological subtypes (acinar, lepidic, micropapillary, papillary, solid). *p*-values were calculated by comparing samples of the corresponding subtype with all other samples using the Wilcoxon rank sum test. *p*-values of significantly higher and lower immune infiltration levels are shown in red and green colors, respectively. The Zabeck dataset GSE58772 [37] was used in this analysis

To compare the TIME among different histological subtypes, we investigated a gene expression dataset for 48 lung adenocarcinoma samples (Zabeck dataset GSE58772 [37]). These samples were carefully prepared by microdissection to obtain pure histology and categorized based on the tumor growth pattern. We applied the BASE computational method [21, 22, 49, 50] to determine the infiltration levels of immune cells in these samples based on their gene expression profiles. Specifically, we calculated the infiltration of six major immune cell types: naïve B cells, memory B cells, CD8+ T cells, CD4+ T cells, NK cells, and myeloid cells. We found that different histological subtypes varied substantially in their immune cell infiltration patterns. Interestingly, lepidic growth pattern tumors tended to have low infiltration in myeloid cells but high infiltration in other immune cell types. In contrast, solid growth pattern tumors demonstrated an opposite trend. As shown, compared with other histological subtypes, lepidic growth pattern tumors had significantly higher infiltration in naïve B cells, memory B cells, CD8+ T cells, and NK cells (*p* < 0.05); solid growth pattern tumors had significantly lower infiltration of these immune cell types but significantly higher infiltration of myeloid cells (Fig. 1; Additional file 2: Fig. S1). Generally, the other three histological subtypes (acinar, papillary, and micropapillary) showed an intermediate level of immune infiltration, with the exception that acinar growth pattern tumors had the lowest infiltration level of NK cells (Fig. 1; Additional file 2: Fig. S1).

Then, we investigated the expression levels of a set of immunostimulatory and immunoinhibitory genes and found that many of these genes were differentially expressed between different histological subtypes. Lepidic growth pattern tumors showed the lowest levels of immune checkpoint B7-H3 (*CD276*) expression (Additional file 2: Fig. S2), which was in line with high infiltration levels of various immune cells in lepidic growth pattern tumors (Fig. 1). Markers of myeloid cells were expressed with a significantly higher level in the solid growth pattern tumors (Additional file 2: Fig. S2), which was consistent with the highest myeloid infiltration level in this histological subtype (Fig. 1). On the contrary, markers associated with immune activation, *CD28* and *CD70*, showed significantly higher expression levels in the lepidic growth pattern tumors (Additional file 2: Fig. S2), which, again, was consistent with the immune cell infiltration results.

### Defining gene signatures to characterize lepidic and solid tumor cells in lung adenocarcinoma samples

**Fig. 2:**
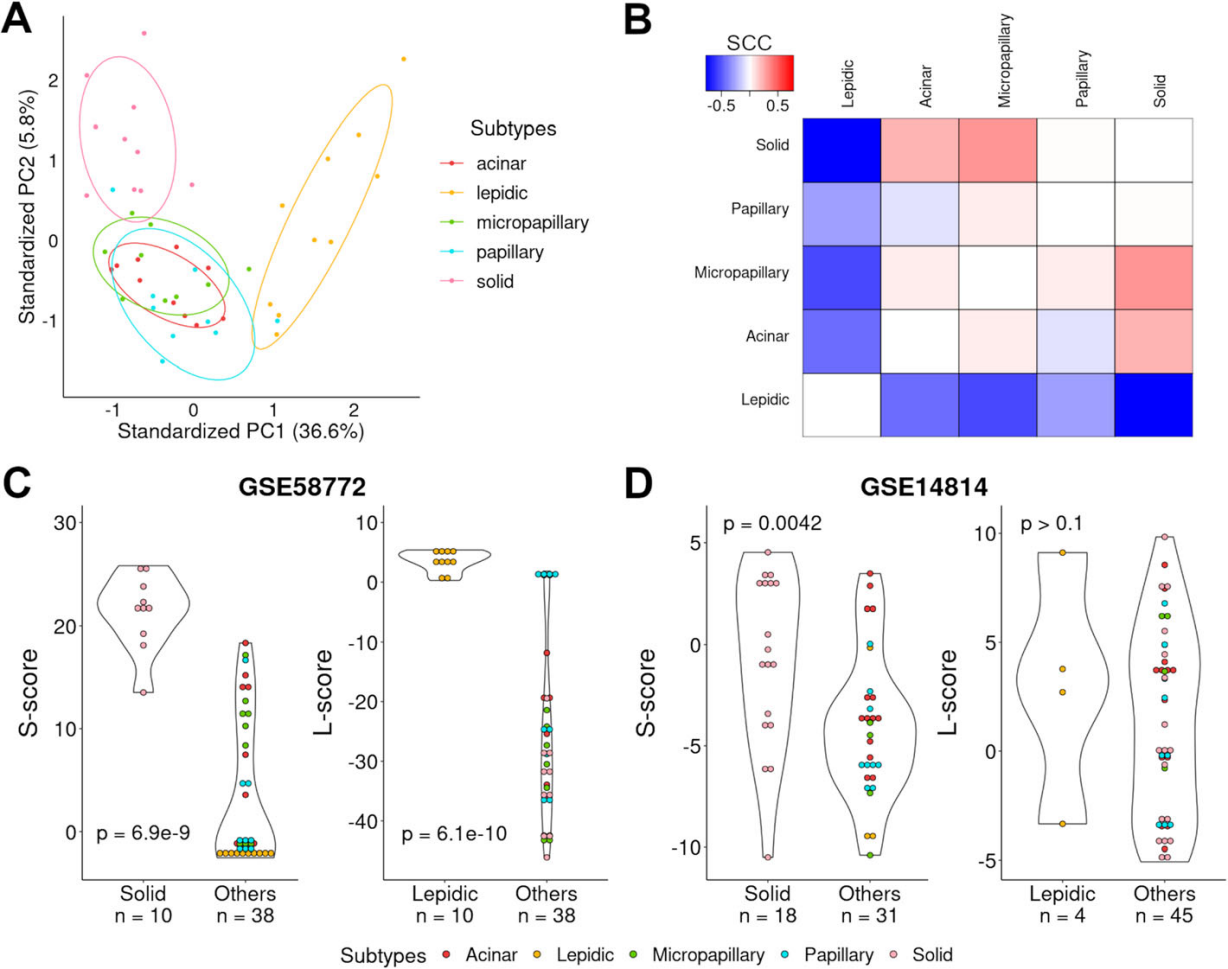
The development of gene signatures for lepidic and solid histological subtypes. **A** PCA plot clustering histological subtypes based on the expression values of 1000 genes. These genes were obtained by pooling the top 200 most specific genes from each of the five subtypes. **B** The Spearman correlation coefficient between the *t*-score profiles for each pair of histological subtypes. **C** The L/S-scores are higher in lepidic/solid-predominant samples than all other samples in the Zabeck dataset GSE58772 [37]. **D** The L/S-scores are higher in lepidic/solid-predominant samples than all other samples in the Zhu dataset GSE14814 [38]. The *p*-values are based on the Wilcoxon rank sum test

We investigated whether gene expression patterns could distinguish different histological subtypes of lung adenocarcinoma. Using the above-described gene expression data from the Zabeck dataset [37], we identified subtype-specific genes by comparing gene expression of each histological subtype with gene expression of all other subtypes using Student’s *t*-test. The significant genes for each subtype (FDR < 0.01) are listed in Additional file 1: Table S2A-E. We identified 7325, 3579, 438, 532, and 244 significant genes (unadjusted *p*-value < 0.005) for the lepidic, solid, acinar, micropapillary, and papillary subtypes, respectively. We noted that no significant genes could be identified to differentiate the papillary subtype from other types after multiple test correction (FDR < 0.05). Thus, the expression patterns of lepidic and solid subtypes are more specific compared with the other subtypes. This result was confirmed by principal component analysis (PCA) based on 1000 selected genes (merging the top 200 most specific genes from each of the five histological subtypes). PCA results revealed distinct clustering of the lepidic and solid subtypes while the acinar, papillary, and micropapillary samples could not be distinguished from each other (Fig. 2A). For each subtype, we obtained a *t*-score profile that characterized its subtype-specific expression pattern. Pairwise correlation analysis between their *t*-score profiles indicated that the lepidic subtype tended to have a negative correlation with the others, especially, the solid subtype while the other four subtypes were positively correlated with each other (Fig. 2B). The same conclusion can be made based on pairwise correlation analysis of gene expression profiles at the sample level (Additional file 2: Fig. S3).

Given these observations, we developed two gene signatures from the gene expression data of the Zabeck dataset [37] to characterize separately the lepidic and solid subtypes. These signatures can be used with gene expression data to determine the relative abundance of lepidic versus solid components (denoted as L-score and S-score, respectively) in lung adenocarcinoma samples. As shown when applied to the Zabeck dataset [37], the lepidic and solid samples showed scientifically higher L-scores and S-scores, respectively, than the other samples (Fig. 2C). This is expected, because the gene signatures for lepidic and solid subtypes are defined based on the same dataset. To further evaluate the two signatures, we applied them to the Zhu dataset GSE14814 [38], which provides histological annotation for 49 lung adenocarcinoma samples. Among these samples, 4 and 18 are annotated as lepidic-predominant and solid-predominant, respectively. In this data, the solid-predominant samples showed significantly higher S-scores than the other samples (*p* = 0.0042, Fig. 2D). Similarly, the lepidic-predominant samples tended to have higher L-scores than the other samples (Fig. 2D) but did not reach statistical significance due to the small sample size (only 4 samples are lepidic-predominant). In addition, we have also validated the signatures in TCGA and CPTAC lung adenocarcinoma datasets [32, 33], in which a small number of samples were annotated as lepidic/solid-predominant (Additional file 2: Fig. S4). These results indicated that the L- and S-scores provided reliable quantifications of the lepidic/solid components in lung adenocarcinoma. In the following analyses, we will focus on L- and S-scores separately, especially the L-score of samples, because the expression profile of the lepidic subtype deviates from and is negatively correlated with those of the other four subtypes.

### High lepidic score is associated with good prognosis

**Fig. 3:**
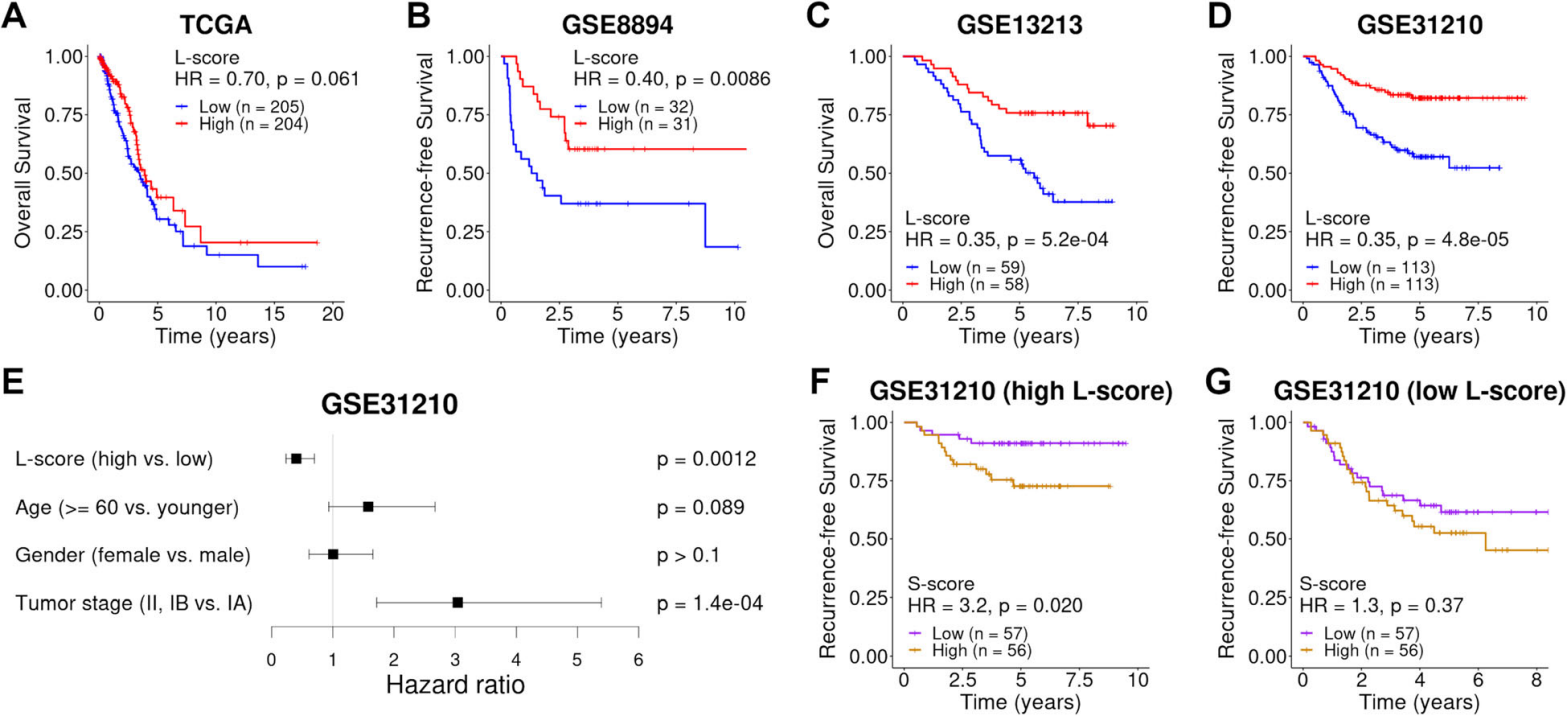
Association of L-scores with patient prognosis in lung adenocarcinoma data. **A**–**D** Kaplan-Meier curves of patients’ survival patterns in the TCGA LUAD [32] (**A**), the GSE8894 [39] (**B**), the GSE13213 [40] (**C**), and the GSE31210 [41] (**D**) dataset. Patients were stratified into two subgroups with high and low L-scores using the median as the cut-off values. **E** Forest plot showing the output from a multivariate Cox regression model for the dataset GSE31210 [41]. The covariates are binary values, including L-score, age, gender, and tumor stage. **F**, **G** Survival curves of the two subgroups stratified based on S-scores in the top 50% of patients with high L-scores (**F**) and the bottom 50% patients with low L-scores (**G**) in the dataset GSE31210 [41]. *p*-values were calculated by log-rank tests, and hazard ratios (HR) were determined by univariate Cox regression models

A large number of lung adenocarcinoma gene expression datasets have been generated in previous studies. For many of them, detailed clinical information was provided, but in most cases, histological composition was not available. The lepidic/solid gene signatures provide a useful tool to investigate the association between lung adenocarcinoma histology and clinical outcomes. To investigate the effect of histological composition on patient prognosis, we calculated patient-specific L/S-scores in four lung adenocarcinoma datasets, including TCGA LUAD [32], GSE8894 [39], GSE13213 [40], and GSE31210 [41]. Using the median value of the L/S-scores, we divided patients into two groups and compared their survival. As shown, patients with high L-scores had significantly prolonged survival compared to patients with low L-scores in all datasets (Fig. 3B–D; Additional file 1: Table S3). Similar results were obtained when L-score = 0 was used to dichotomize patient samples (Additional file 1: Table S3). Note that either overall or recurrence-free survival was used depending on the availability in different datasets.

Moreover, multivariate Cox regression analysis indicated that the L-score was still significant after considering well-established clinical factors, including age, gender, and tumor stage (Fig. 3E). When the L-score was used as a continuous variable, a more significant association between L-score and prognosis was observed in both univariate and multivariate Cox regression analyses (Additional file 1: Table S3). Similarly, we performed the same analyses for the solid signature and found that the resulting S-score was also predictive of patient prognosis in lung adenocarcinoma, with higher S-scores associated with worse prognosis (Additional file 1: Table S3).

Furthermore, our results showed that combining the L-score with the S-score could further improve prognostic prediction. In Fig. 3F, we selected patients with high L-scores and further divided them into two subgroups with high and low S-scores, respectively. As shown, significantly differential survival was observed between these two subgroups. No similar pattern was found in the patient subset with low L-scores (Fig. 3G). Nevertheless, these results indicated that the L-score and S-score provided related but complementary information for prognosis prediction.

### Association of lepidic and solid scores with genomic features

**Fig. 4:**
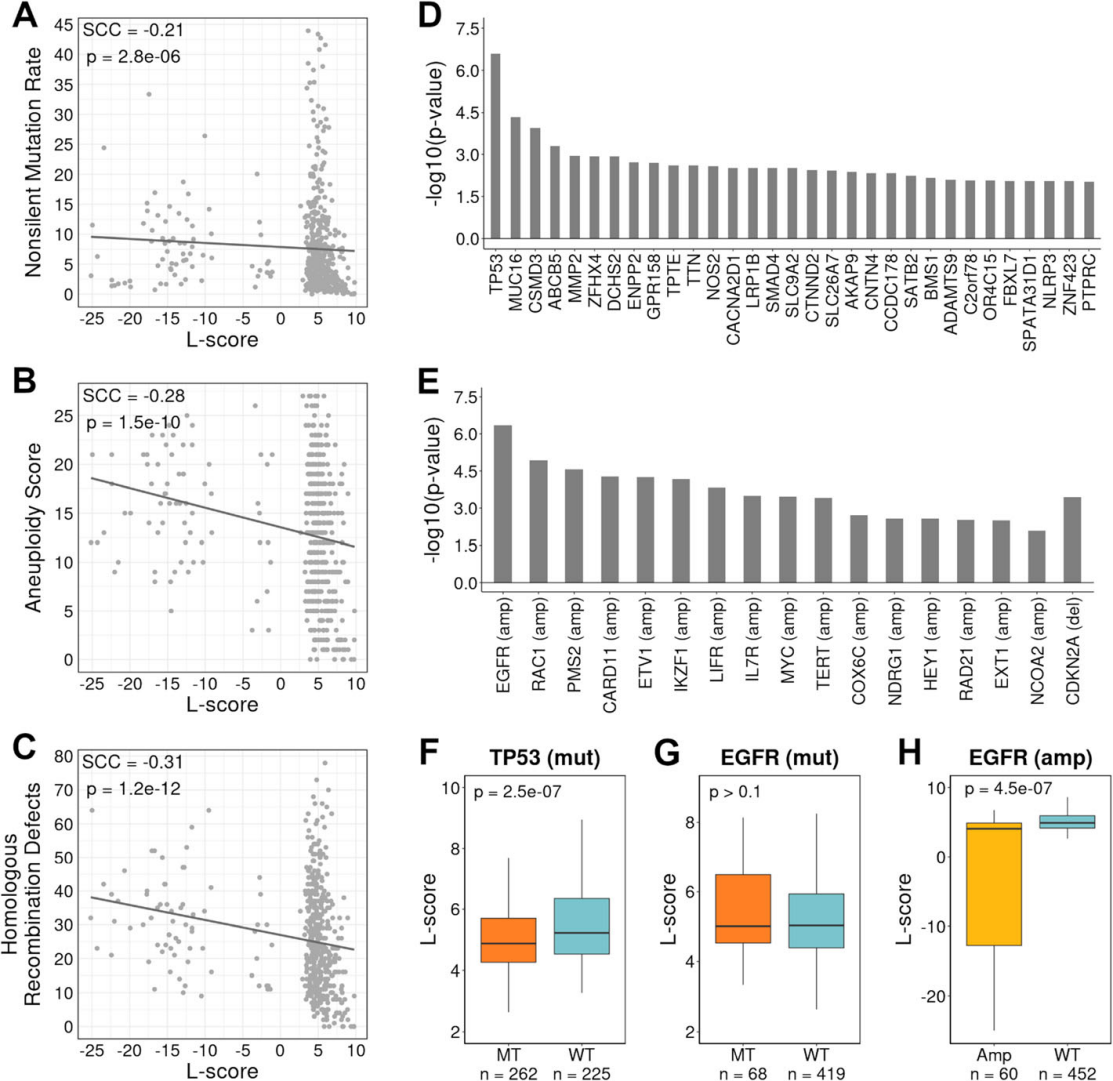
Associations between L-scores and genomic features. **A**–**C** The correlation between L-score and non-silent mutation rate (**A**), aneuploidy score (**B**), and homologous recombination defects (**C**), respectively. **D** Genes with mutation status significantly correlated with the L-score of samples. **E** Genes with amplification/deletion status significantly correlated with the L-score of samples. **F** *TP53* mutant samples show significantly lower L-scores than wild-type samples. **G** No significant correlation between *EGFR* mutation status and L-scores. **H** *EGFR* amplified samples show significantly lower L-scores than wild-type samples. In **D**–**H**, *p*-values were calculated by using the Wilcoxon rank sum test. SCC Spearman correlation coefficient

To gain insight on what genomic features might have an impact on the histological composition in lung adenocarcinoma, we examined the association between L-score and several genomic features using TCGA LUAD [32]. We found that L-score was negatively correlated with tumor mutation burden (TMB), represented by non-silent mutation rate (SCC = −0.21, *p* = 2.8e−06, Fig. 4A). The L-score was also negatively correlated with tumor aneuploidy score, which was calculated as the sum of altered arms (SCC = −0.28, *p* = 1.5e−10, Fig. 4B). The aneuploidy score reflects the degree of chromosome instability, which is associated with the deficiency of the homologous recombination pathway. Consistently, the L-score was also significantly correlated with the deficiency of this pathway (SCC = −0.31, *p* = 1.2e−12, Fig. 4C). In contrast, similar analyses with S-score and the genomic features showed positive correlations. In particular, S-score was positively correlated with TMB (SCC = 0.35, *p* = 1.1e−15, Additional file 2: Fig. S5A), with tumor aneuploidy score (SCC = 0.28, *p* = 2.1e−10, Additional file 2: Fig. S5B), and with homologous recombination pathway deficiency (SCC = 0.41, *p* < 2.2e−16, Additional file 2: Fig. S5C).

Following that, we identified genes whose mutation or gain/loss was correlated with the samples’ L-scores. In total, the mutation statuses of 32 genes were significantly correlated with L-scores. All of them except for *MAGI2* had lower L-scores in mutated samples than the corresponding wild-type samples (Fig. 4D; Additional file 1: Table S4A). In other words, samples with mutations in these genes tend to have a lower lepidic composition in tumors. This result aligns with the association of L-scores with a good prognosis as described in the previous section. In addition, samples with amplification in 16 genes had higher L-scores than wild-type samples, and samples with *CDKN2A* deletion had higher L-scores than wild-type samples (Fig. 4E; Additional file 1: Table S4B). We performed the analyses with S-score and identified the mutation status of 279 genes, as well as the amplification status of 12 genes which were significantly correlated with S-scores (Additional file 1: Table S4C-D). There are no genes whose deletion status was significantly correlated with S-scores.

The mutation status of the *TP53* gene was significantly correlated with L-scores, with mutated samples being associated with lower L-scores compared to wild-type samples (*p* = 2.5e−07, Fig. 4D; Fig. 4F; Additional file 1: Table S4A). *TP53* plays critical roles in tumorigenesis [53] and its mutation correlates with poor prognosis in many cancer types [54–56]. *EGFR* is another important driver gene in lung cancer with multiple drugs targeting its protein products (EGFR inhibitors) being approved by the FDA [57–59]. The somatic mutation status of *EGFR* was not correlated with the L-score (*p* > 0.1, Fig. 4G). However, samples with *EGFR* amplification showed significantly lower L-scores than wild-type samples (*p* = 4.5e−07, Fig. 4H; Additional file 1: Table S4B). In fact, it is the most significant among the 16 genes with gain/loss status correlating with L-score in lung adenocarcinoma (Fig. 4E). To further verify this result, we combined *EGFR* mutation and amplification information to divide patients into four groups: with only mutation (mut + amp-), with only amplification (amp + mut-), with both (amp + mut+), and with neither (wt). Patients with only mutated *EGFR* (mut + amp-) had similar L-scores with the wild-type patients (wt) (*p* > 0.1, Additional file 2: Fig. S6). In contrast, patients with amplified *EGFR* had significantly lower L-scores than the wild-type patients with *p* = 5.1e−05 for amp + mut- and *p* = 0.0022 for amp + mut + with respect to the wt (Additional file 2: Fig. S6). These results confirmed that a reduced L-score is associated with *EGFR* amplification but not *EGFR* mutation.

In addition, we observed the opposite patterns for the association of S-scores with these genes. Samples with mutated *TP53* had higher S-scores than wild-type samples (*p* = 4.6e−24, Additional file 1: Table S4C; Additional file 2: Fig. S5D), and while *EGFR* mutation did not have a significant association with S-score (*p* > 0.1, Additional file 2: Fig. S5E), samples with amplified *EGFR* had higher S-scores than wild-type samples with the most significance (*p* = 2.3e−05, Additional file 1: Table S4D; Additional file 2: Fig. S5F). These results suggest that tumor histology may not just be simply affected by deregulated oncogenic pathways (e.g., overactivation of the EGFR pathway), rather, the molecular status of the driver gene product (e.g., abnormal structure vs. elevated abundance of the EGFR proteins) might also be important.

### Association of lepidic and solid scores with lung cancer cell line sensitivity to targeted drugs

**Fig. 5:**
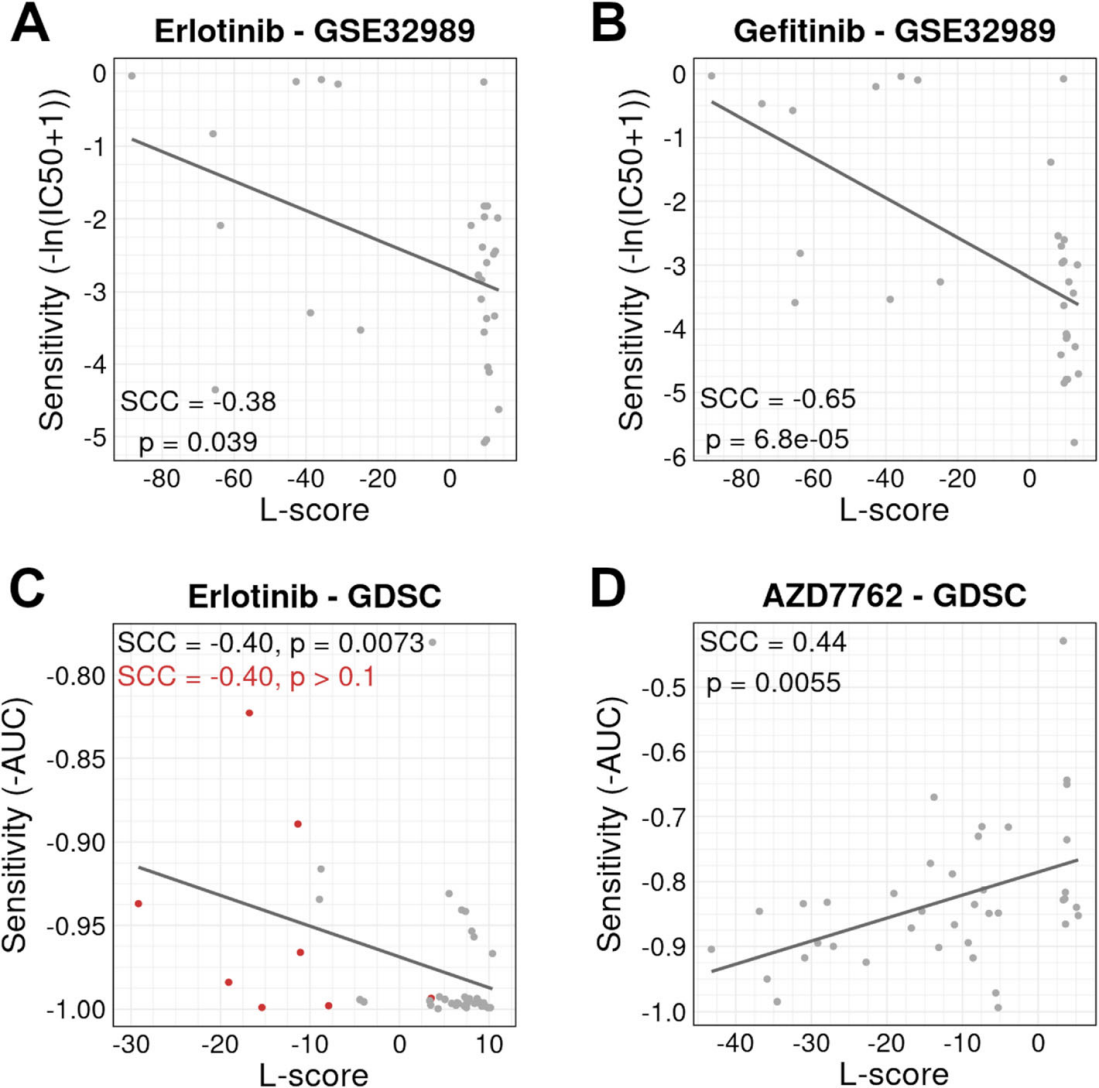
Correlations between L-scores and drug sensitivity in lung cancer cell lines. **A**, **B** The L-score is negatively correlated with cell sensitivity to EGFR inhibitors, erlotinib (**A**) and gefitinib (**B**) in the dataset GSE32989 [42]. **C** The L-score is negatively correlated with cell sensitivity to erlotinib in the dataset GDSC [35]. The association is not significant in lung adenocarcinoma cell lines due to the low sample number with sensitivity data (red) but is significant in all lung cancer cell lines (gray). **D** The L-score is positively correlated with cell sensitivity to AZD7762 in the dataset GDSC [35]. Drug sensitivity is represented as -ln(IC50 + 1) in GSE32989 [42] and as -AUC (area under the curve) in GDSC [35]. SCC Spearman correlation coefficient

Knowing that L-score was negatively correlated with *EGFR* amplification status in lung adenocarcinoma, we then examined the association between L-score and lung adenocarcinoma cell line sensitivity to EGFR inhibitors. The GSE32989 data provides the baseline gene expression profiles of 31 lung adenocarcinoma cell lines as well as their sensitivity (IC50) to two EGFR inhibitors (erlotinib and gefitinib) [42]. In this data, we observed a significantly negative correlation between L-score and erlotinib sensitivity (SCC = −0.38, *p* = 0.039, Fig. 5A) and between L-score and gefitinib sensitivity (SCC = −0.65, *p* = 6.8e−05, Fig. 5B).

We then examined the Genomics of Drug Sensitivity in Cancer (GDSC) data, which provide cell line sensitivity information for a total of 138 different drugs (including erlotinib) in 714 cancer cell lines, including 124 lung cancer cell lines (42 are lung adenocarcinoma cell lines) [35]. In the data, we confirmed the correlation between L-score and erlotinib sensitivity in lung adenocarcinoma cell lines with SCC = −0.40 (Fig. 5C), although statistical significance was not reached due to lack of statistical power (the IC50 value was only available for only 8 cell lines). When all lung cancer cell lines were used, we observed a significant correlation with SCC = −0.40 and *p* = 0.0073.

In addition to EGFR inhibitors, our results indicated that the L-score was correlated with lung cancer cell line sensitivity to other drugs tested in the GDSC data. For example, AZD7762, a CHK1 inhibitor, presented a significant association with L-score (SCC = 0.44, *p* = 0.0055, Fig. 5D), suggesting that lung tumors with higher L-scores might be more responsive to this drug. CHK1 pathway regulating DNA damage and cell cycle responses is suggested as one of the causes of treatment resistance in lung cancer and is currently an emerging target for therapy [60–62]. Similar analyses were performed for the S-signature and identified drugs correlated with S-scores in lung cancer. For example, lung cancer cell line sensitivity to EHT 1864 (a RAC inhibitor) was correlated with S-score (SCC = −0.47, *p* = 0.0027, Additional file 2: Fig. S7). Inhibiting the RAC pathway has been recommended as an alternative option for treating EGFR inhibitor-resistant patients with lung cancer [63, 64].

### L-score is predictive of patient sensitivity to immunotherapy in lung cancer

**Fig. 6:**
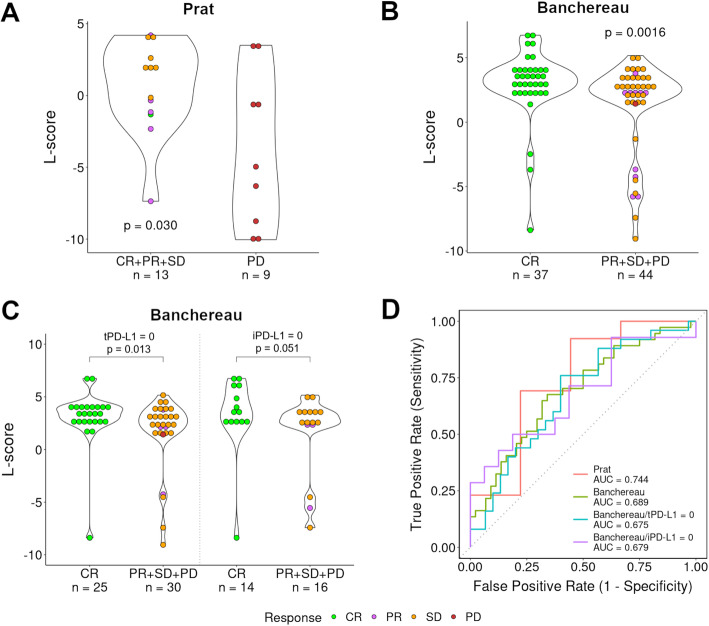
Association of the L-score with patient response to immunotherapy in lung cancer. The Prat and Banchereau datasets [43, 47] were used in this analysis, and patients were divided into two groups based on their response to immunotherapy. Lung adenocarcinoma patients were separated into responders and non-responders in the Prat cohort [43]. Responders include 1 patient with complete response (CR), 5 with partial response (PR), and 7 with stable disease (SD). Non-responders are 9 patients with progressive disease (PD). In the Banchereau cohort [47], non-small cell lung cancer patients were divided into two groups: 37 patients with CR and 44 patients with other responses—10 with PR, 33 with SD, and 1 with PD. **A** Responders (1CR + 5PR + 7SD) showed significantly higher L-scores than non-responders (9PD) in the Prat cohort [43]. **B** Responders (37CR) showed significantly higher L-scores than non-responders (10PR + 33SD + 1PD) in the Banchereau cohort [47]. Of note, the responder/non-responder groups were defined differently between the two datasets in order to balance group sizes. **C** The association of the L-score with patient response in Banchereau cohort [47] patients with no PD-L1 protein expression in tumor or immune cells. **D** Receiver operating characteristic (ROC) curves with the L-score as a predictor of patient response. CR complete response, PR partial response, SD stable disease, PD progressive disease, tPD-L1 PD-L1 protein expression in tumor cells, iPD-L1 PD-L1 protein expression in immune cells

As we have found that different histological subtypes varied dramatically in the immune microenvironment, we investigated the relationship between L-score and patient response to immunotherapy. We retrieved two datasets that contained gene expression profiles and treatment outcome information for lung cancer patients. The Prat dataset (GSE93157) consists of 22 adenocarcinoma and 13 squamous cell carcinoma treated with pembrolizumab and nivolumab [43]. The Banchereau dataset consists of 81 non-small cell lung cancer treated with atezolizumab [47]. We calculated the L-scores of these patients based on their gene expression data. Based on the treatment outcomes, we divided the lung cancer patients into responders and non-responders. We observed a significantly higher L-score in the responder group compared to the non-responder group in both the Prat cohort [43] (*p* = 0.030, Wilcoxon rank sum test, Fig. 6A) and the Banchereau cohort [47] (*p* = 0.0016, Wilcoxon rank sum test, Fig. 6B). The PD-L1 protein expression in tumor (tPD-L1) or immune cells (iPD-L1) has been measured in the Banchereau dataset [47]. We found that in patients with no PD-L1 expression the L-score was predictive of patient response with *p* = 0.013 for the tPD-L1 = 0 group and *p* = 0.051 for the iPD-L1 = 0 group (Fig. 6C). To quantify the ability of using an L-score to classify responders vs. non-responders, we determined their receiver operating characteristic (ROC) curves and the area under the curve (AUC). The L-score could classify the patient groups with AUC = 0.744 in the Prat cohort [43] (Fig. 6D), AUC = 0.689 for all (*n* = 81), AUC = 0.675 for tPD-L1 = 0 (*n* = 55), and AUC = 0.679 for 30 iPD-L1 = 0 NSCLC samples in the Banchereau cohort [47] (Fig. 6D). Furthermore, multivariate logistic regression analysis indicated the predictive value of L-score for patient response after considering established clinical factors, including age, sex, and *CD274* expression level (Additional file 1: Table S5). In contrast with L-score, we did not observe a significant association between S-score and immunotherapy response in both datasets.

## Discussion

In this study, we identified significantly differential immune cell infiltration between the five different histological subtypes of lung adenocarcinoma using a histology-specific gene expression data from micro-dissected tumor samples. We found that the lepidic and solid subtypes could be determined based on their gene expression patterns, while the other three subtypes (acinar, micropapillary, and papillary) had similar expression patterns. Motivated by these observations, we defined two gene signatures and used them to determine the relative abundance of lepidic and solid tumor components in lung adenocarcinoma samples. We found that L-scores and S-scores were significantly associated with patient prognosis and with genomic features such as TMB in lung adenocarcinoma. In particular, the L-scores of lung cancer cell lines were negatively correlated with their sensitivity to EGFR inhibitors. Moreover, using an immunotherapy cohort data, we found that the L-score was significantly associated with patient response to ICBT in lung cancer.

Consistent with previous studies, our results indicated that samples with higher L-scores (indicating higher proportion of lepidic histology) tended to have a better prognosis, whereas higher S-scores were associated with a worse prognosis. These results might be explained at least partially by results from our immune infiltration analysis. The lepidic subtype had the highest levels of B cells, CD8+ T cells, and NK cells, which are known to play positive roles during immunosurveillance, whereas the immunosuppressive myeloid cells had the lowest infiltration. In contrast, the prognostically unfavorable solid subtype displayed the opposite pattern in immune cell infiltration.

It is well known that distinct histological subtypes are associated with different mutation landscapes in tumor cells [65, 66]. However, it remains unclear how genomic alterations of driver genes determine the tumor histology, and vice versa. In this study, we determined the significant association of L- and S-scores with tumor mutation burden and *TP53* mutation status, as well as poor association with *EGFR* mutation status. We validated our results in the immunotherapy response dataset Banchereau [47] and three independent GEO datasets: GSE11969 [44], GSE26969 [45], and GSE72094 [46] (Additional file 1: Table S6). Interestingly, in this study, we found that *EGFR* amplification but not *EGFR* mutation was significantly correlated with the L-score and S-score of tumors. Both amplification and mutation of *EGFR* are frequently observed in lung cancers, which lead to the overactivation of the EGFR singling pathway. *EGFR* amplification results in enhanced protein abundance on the membrane of tumor cells, while *EGFR* mutation gives rise to abnormal protein products. It seems that, at least in this case for *EGFR*, the oncogenic pathways associated with more protein product has a more substantial impact on histology compared with effects from an abnormal protein.

Initially, we planned to develop a deconvolution-based method to calculate the proportion of the five histological subtypes in lung adenocarcinoma samples. In fact, we have attempted to do this by using the CIBERSORT [67] and WISP [68] algorithms. However, none of them could generate effective reference signatures for all five histological subtypes. Specifically, the gene expression patterns of acinar, papillary, and micropapillary subtypes were similar. No subtype-specific genes can be identified using the Zabeck dataset [37] after correcting multiple tests. As such, in this study, we focused on the lepidic and solid subtypes, which had expression patterns distinguishable from the other subtypes. Instead of calculating the histology proportion, we defined two gene signatures to quantify the relative abundance of lepidic and solid subtypes of tumor cells, respectively.

We showed here that a higher L-score was associated with low sensitivity to EGFR inhibitors in lung cancer cell lines. However, the predictive value of the L-signature was not validated in lung adenocarcinoma patients treated with EGFR inhibitors due to the lack of appropriate datasets. Additionally, we showed that this signature is predictive of patient response to immunotherapy in the Prat and the Banchereau datasets [43, 47]. As another caveat, we used all NSCLC samples in the Banchereau cohort [47], since the histological types (adenocarcinoma or squamous) of the tumor samples were not provided. According to our results, we expect that both EGFR-targeted therapy and immunotherapy might change the histological composition of lung adenocarcinoma. Specifically, the tumor subclones of lepidic subtypes are likely to survive from EGFR-targeted treatment but may be killed by immunotherapy. We have not found data to perform this analysis, but it would be interesting to examine how the L-score changes before and after therapeutic treatment when data become available.

The current L/S-signature is a whole gene signature that includes all genes with weights assigned according to their subtype specificity. The signature can be further simplified by choosing the top most informative (i.e., highly weighted) genes to facilitate its clinical application. The L/S-scores indicate the relative abundance of L/S-subtype tumor cells, but do not automatically identify subtype predominance. To identify L/S-predominant tumor samples, the scores can be compared with reference scores that are produced for L/S-specific reference samples using the same gene expression platform.

## Conclusions

In conclusion, we have defined gene signatures to quantify the relative abundance of lepidic and solid subtypes in lung adenocarcinoma. By using these signatures, we detected important associations between the L/S-score and clinical outcomes, which suggested potential clinical translation. The framework developed in this study can also be applied to other cancer types with heterogeneous subtypes.

## Supplementary Information

**Additional file 1: Table S1-S7.**

**Additional file 2: Figs. S1-S7.**

## Data Availability

The Cancer Genome Atlas (TCGA) data of lung adenocarcinoma (LUAD) are available from FireBrowse (http://firebrowse.org) [32]. Some genomic features of TCGA LUAD samples are available from a published article by Thorsson et al. [48]. LUAD sample data from The National Cancer Institute’s (NCI’s) Clinical Proteomic Tumor Analysis Consortium (CPTAC) are available from NCI’s Genomic Data Commons (GDC) [33, 34]. The Genomics of Drug Sensitivity in Cancer (GDSC) data are available from the GDSC database [35]. Data from Gene Expression Omnibus (GEO) [36] are available with accession numbers GSE58772 [37], GSE14814 [38], GSE8894 [39], GSE13213 [40], GSE31210 [41], GSE32989 [42], GSE93157 [43], GSE11969 [44], GSE26939 [45], and GSE72094 [46]. Data from Banchereau et al. [47] are available from The European Genome-phenome Archive, accession number EGAS00001004343.

## Abbreviations

NSCLC: Non-small cell lung cancer
ICBT: Immune checkpoint blockade therapy
TIME: Tumor immune microenvironment
LUAD: Lung adenocarcinoma
TCGA: The Cancer Genome Atlas
NCI: National Cancer Institute
CPTAC: Clinical Proteomic Tumor Analysis Consortium
GDC: Genomic Data Commons
GDSC: The Genomics of Drug Sensitivity in Cancer
GEO: Gene Expression Omnibus
PCA: Principal component analysis
GSEA: Gene set enrichment analysis
FDR: False discovery rate
TMB: Tumor mutation burden
SCC: Spearman correlation coefficient
AUC: Area under the curve
ROC: Receiver operating characteristic
CR: Complete response
PR: Partial response
SD: Stable disease
PD: Progressive disease
tPD-L1: PD-L1 protein expression in tumor cells
iPD-L1: PD-L1 protein expression in immune cells
