# Supplementary material for: A lepidic gene signature predicts patient prognosis and sensitivity to immunotherapy in lung adenocarcinoma
Source: Genome Med. 2022 Jan 12;14:5. doi: 10.1186/s13073-021-01010-w (PMC8753834; doi:10.1186/s13073-021-01010-w)
Supplement: Supplementary file 2 — Additional file 2: Figs. S1-S7. [file 13073_2021_1010_MOESM2_ESM.pdf]

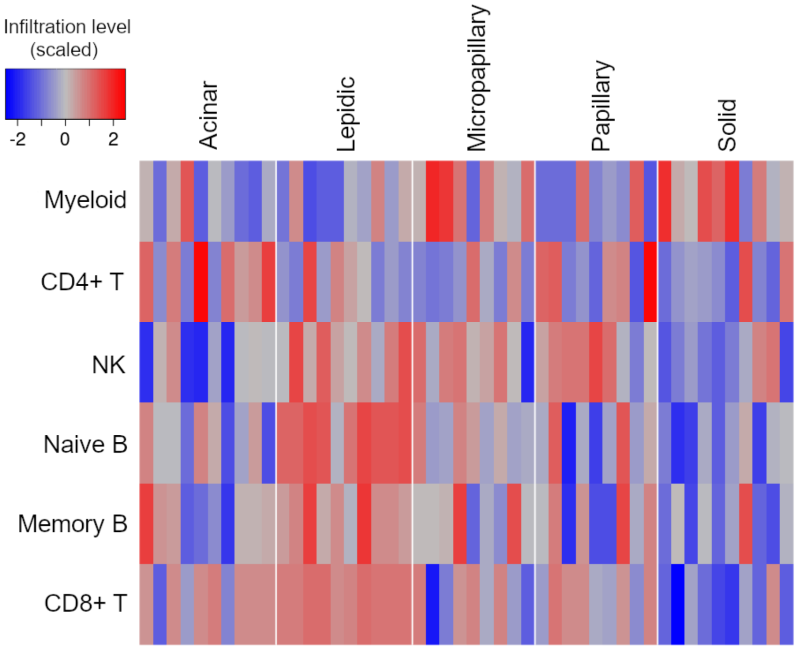

Fig. S1: The infiltration levels of immune cells vary significantly between different histological subtypes of lung adenocarcinoma. Scaled heatmap showing the infiltration levels of Myeloid, CD4+ T, NK, Naïve B, Memory B, CD8+ T in five different lung adenocarcinoma histological subtypes (acinar, lepidic, micropapillary, papillary, solid). The Zabeck dataset GSE58772 [1] was used in this analysis.

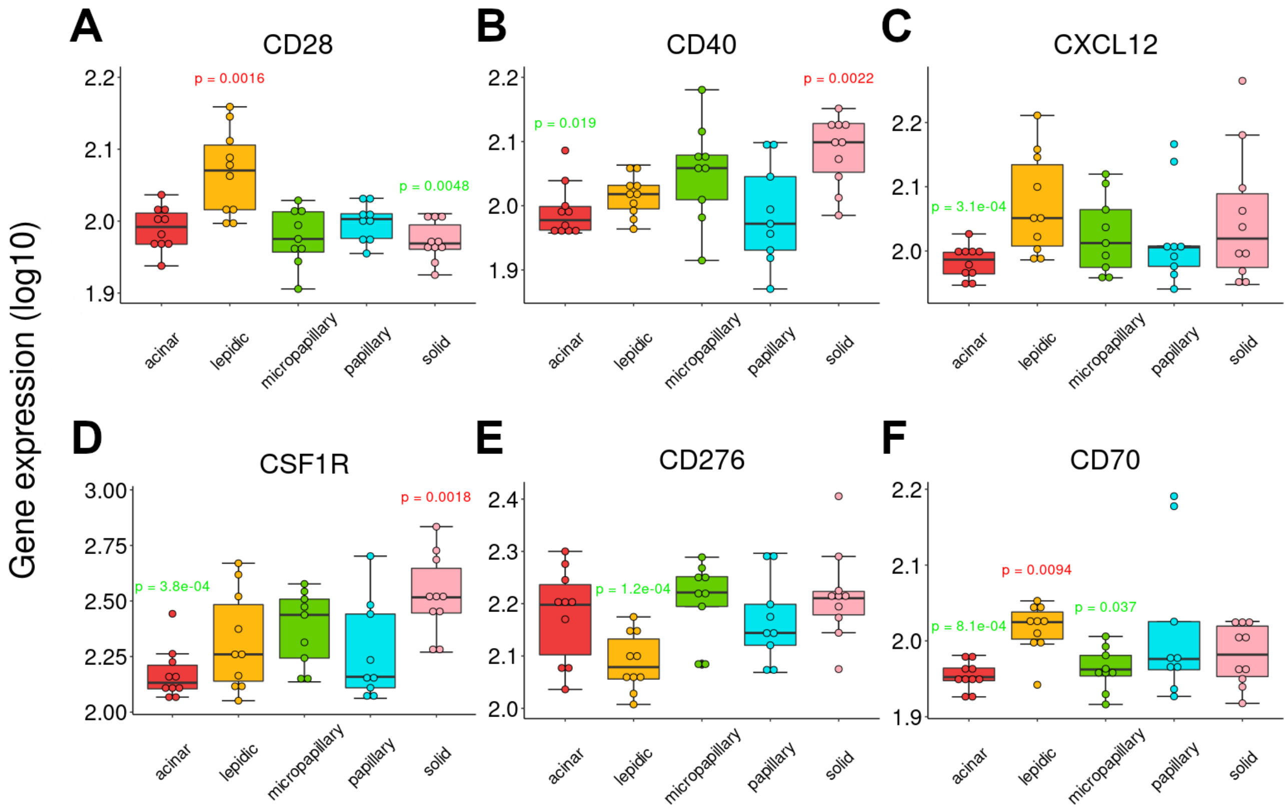

Fig. S2: Immune-related genes are differentially expressed between histological subtypes of lung adenocarcinoma. Boxplots showing expression levels in log base 10 of (A) *CD28*, (B) *CD40*, (C) *CXCL12*, (D) *CSF1R*, (E) *CD276*, and (F) *CD70* in five different lung adenocarcinoma histological subtypes (acinar, lepidic, micropapillary, papillary, solid). P-values were calculated by comparing samples of the corresponding subtype with all other samples using the Wilcoxon rank sum test. P-values of significantly higher and lower are shown in red and green color, respectively. The Zabeck dataset GSE58772 [1] was used in this analysis.

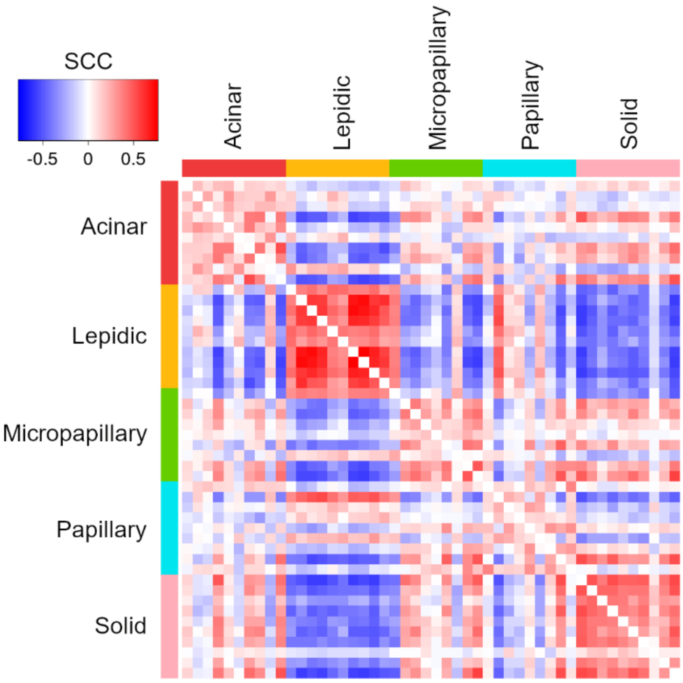

Fig. S3: Lepidic subtype is negatively correlated with four other subtypes and, specifically, highly correlated with solid subtype. Heatmap showing the Spearman correlation coefficient between the gene expression profiles for each pair of samples. The gene expression profiles were obtained by pooling the top 200 most specific genes from each of the five subtypes. The five subtypes (acinar, lepidic, micropapillary, papillary, solid) are color labeled as red, orange, green, blue, pink, respectively. The Zabeck dataset GSE58772 [1] was used in this analysis.

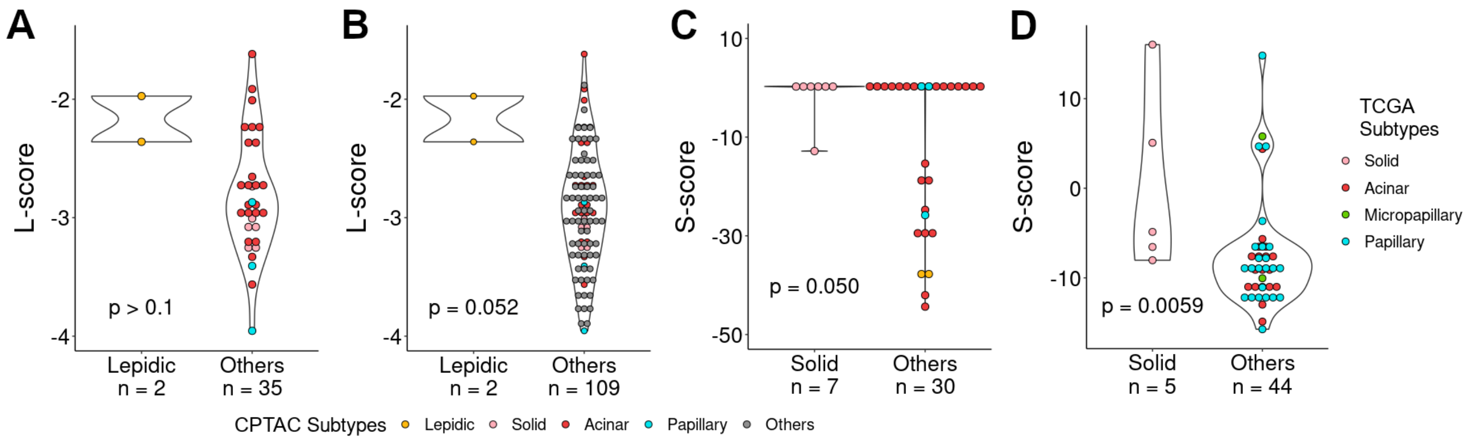

Fig. S4: Comparison of L- and S-scores between lepidic/solid predominant subtypes and other subtypes in CPTAC and TCGA [2, 3]. **A-B**: L-scores were higher but not statistically significant in lepidic predominant samples compared to other samples with predominant histology (A), but significant compared to all other samples in CPTAC [2] (B). **C-D**: S-scores were higher in solid predominant samples than in other subtype-predominant samples in CPTAC [2] (C) and in TCGA [3] (D). P-values were calculated by using the Wilcoxon rank sum test.

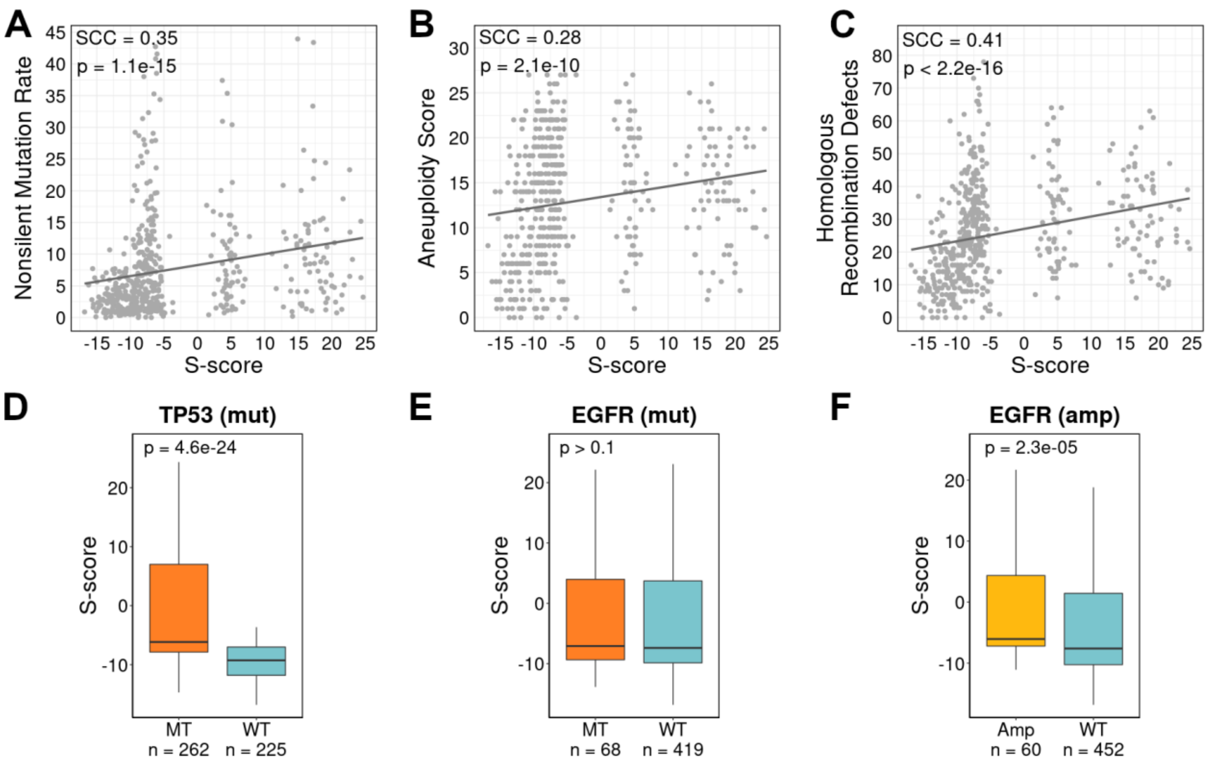

Fig. S5: Associations between S-scores and genomic features. **A-C**: The correlation between S-score and non-silent mutation rate (A), aneuploidy score (B), and homologous recombination defects (C), respectively. **D**: *TP53* mutant samples show significantly higher S-scores than wild-type samples. **E**: No significant correlation between *EGFR* mutation status and S-scores. **F**: *EGFR* amplified samples show significantly higher S-scores than wild-type samples. In D-F, P-values were calculated by using the Wilcoxon rank sum test. SCC: Spearman correlation coefficient.

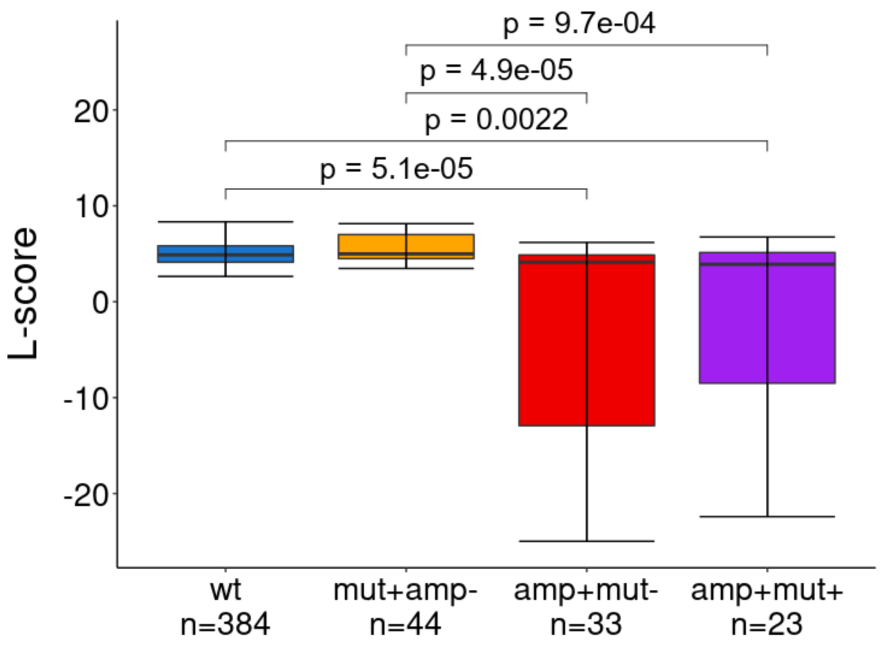

Fig. S6: Comparison of L-score based on EGFR mutation/amplification statuses in TCGA [3]. Patients were divided into four groups: no mutation and amplification (wt), with only mutation (mut+amp-), with only amplification (amp+mut-), and with both (amp+mut+). P-values were calculated by using the Wilcoxon rank sum test.

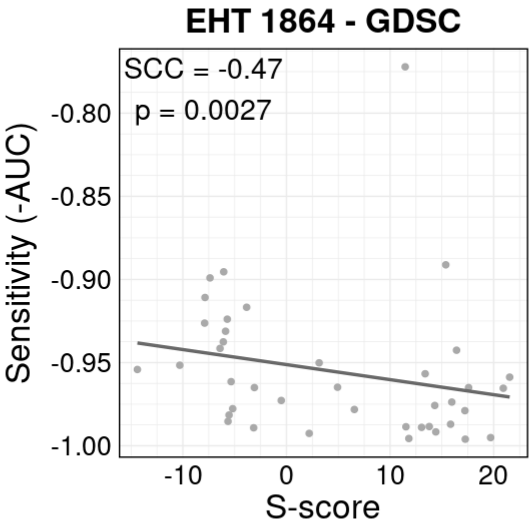

Fig. S7: S-score is positively correlated with cell sensitivity to EHT 1864 in the dataset GDSC [4]. Drug sensitivity is represented as -AUC (Area Under Curves). SCC: Spearman correlation coefficient.

References:

1. Zabeck H, Dienemann H, Hoffmann H, Pfannschmidt J, Warth A, Schnabel PA, et al. Molecular signatures in IASLC/ATS/ERS classified growth patterns of lung adenocarcinoma. PLoS One. 2018;13:e0206132. doi:10.1371/journal.pone.0206132.

2. Edwards NJ, Oberti M, Thangudu RR, Cai S, McGarvey PB, Jacob S, et al. The CPTAC Data Portal: A Resource for Cancer Proteomics Research. J Proteome Res. 2015;14:2707–13. doi:10.1021/pr501254j.

3. Cancer Genome Atlas Research Network. Comprehensive molecular profiling of lung adenocarcinoma. Nature. 2014;511:543–50. doi:10.1038/nature13385.

4. Yang W, Soares J, Greninger P, Edelman EJ, Lightfoot H, Forbes S, et al. Genomics of Drug Sensitivity in Cancer (GDSC): a resource for therapeutic biomarker discovery in cancer cells. Nucleic Acids Res. 2012;41:D955–61. doi:10.1093/nar/gks1111.
